# Holding Biological Motion in Working Memory: An fMRI Study

**DOI:** 10.3389/fnhum.2016.00251

**Published:** 2016-06-01

**Authors:** Xiqian Lu, Jian Huang, Yuji Yi, Mowei Shen, Xuchu Weng, Zaifeng Gao

**Affiliations:** ^1^Department of Psychology, Zhejiang UniversityHangzhou, China; ^2^Network Center, Women’s Hospital, School of Medicine, Zhejiang UniversityHangzhou, China; ^3^Department of Speech and Hearing Science, Arizona State University, TempeAZ, USA; ^4^Center for Cognition and Brain Disorders, Hangzhou Normal UniversityHangzhou, China

**Keywords:** biological motion, working memory, point-light display, mirror neuron system, fMRI

## Abstract

Holding biological motion (BM), the movements of animate entities, in working memory (WM) is important to our daily life activities. However, the neural substrates underlying the WM processing of BM remain largely unknown. Employing the functional magnetic resonance imaging (fMRI) technique, the current study directly investigated this issue. We used point-light BM animations as the tested stimuli, and explored the neural substrates involved in encoding and retaining BM information in WM. Participants were required to remember two or four BM stimuli in a change-detection task. We first defined a set of potential brain regions devoted to the BM processing in WM in one experiment. We then conducted the second fMRI experiment, and performed time-course analysis over the pre-defined regions, which allowed us to differentiate the encoding and maintenance phases of WM. The results showed that a set of brain regions were involved in encoding BM into WM, including the middle frontal gyrus, inferior frontal gyrus, superior parietal lobule, inferior parietal lobule, superior temporal sulcus, fusiform gyrus, and middle occipital gyrus. However, only the middle frontal gyrus, inferior frontal gyrus, superior parietal lobule, and inferior parietal lobule were involved in retaining BM into WM. These results suggest that an overlapped network exists between the WM encoding and maintenance for BM; however, retaining BM in WM predominately relies on the mirror neuron system.

## Introduction

Processing biological motion (BM), the movements of animate entities (Johansson, 1973; see Troje, 2013 for a review), is vital to daily life activities such as social interaction, motor learning, and non-verbal communication (for reviews, see Blake and Shiffrar, 2007; Steel et al., 2014). To process BM information, our visual system has evolved a sophisticated mechanism that can be demonstrated clearly via point-light displays (PLDs; Johansson, 1973). PLDs isolate human kinematics by depicting human activity via a few (e.g., 12) light points. Although highly impoverished, once in motion, these PLDs can be rapidly recognized as coherent, meaningful movements. Moreover, multiple aspects of social information can be extracted from PLDs, including walking direction, gender, interactions with other people, and emotion, even if the PLDs are presented within random dynamic noise (for reviews, see Puce and Perrett, 2003; Blakemore, 2008; Troje, 2013; Steel et al., 2014). However, to form coherent BM representations in the mind and guide our social behavior, we need to not only process the BM in perception but also retain the BM information in *working memory* (WM; e.g., Wood, 2007, 2011; Shen et al., 2014; Gao et al., 2015), which stores and manipulates a limited set of information (Baddeley and Hitch, 1974; Baddeley, 2012). Indeed, it has been suggested that the WM capacity of BM, but not other stimuli (e.g., colors, moving rectangles), is positively correlated with one’s empathy level (Gao et al., 2016). Therefore, it is important to elucidate the processing mechanisms of BM in WM.

Currently, there have been few studies exploring the mechanisms of retaining BM in WM. Most of these studies focused on the storage mechanisms of BM and BM-related bindings in WM. It has been demonstrated that BM has a storage buffer in WM that is independent from location, color, and shape, and can retain 3–4 BM stimuli (Smyth et al., 1988; Smyth and Pendleton, 1989, 1990; Wood, 2007, 2011; Shen et al., 2014). However, storing BM-related bindings in WM is quite resource-exhausting; for instance, only 1–2 BM-color bindings can be held in WM (Wood, 2008; Ding et al., 2015). Moreover, in contrast to robust object-based encoding in WM for common visual objects, in which irrelevant information is involuntarily encoded with relevant information (e.g., both shape and color when only the color of colored shapes is required to be stored in WM), there is no object-based encoding for colored BM stimuli (Ding et al., 2015). However, all the aforementioned studies have addressed BM processing in WM via behavioral methods. Thus, the neural mechanisms underlying WM processing of BM remain largely unknown.

To the best of our knowledge, only one study has examined the neural mechanisms of BM processing in WM. Gao et al. (2015) investigated whether the mirror neuron system (MNS) was involved in retaining BM information in WM using PLDs as stimuli. The MNS refers to a neural network that includes the ventral premotor cortex (vPMC), posterior inferior frontal gyrus (pIFG), and rostral inferior parietal lobule (rIPL; e.g., Saygin et al., 2004; Van Overwalle and Baetens, 2009; Grosbras et al., 2012; Thompson and Parasuraman, 2012). The MNS has a “mirror” mechanism in the sense that the same set of neurons is activated both when a person performs an action and when he/she observes another person performing the same action (e.g., Rizzolatti and Craighero, 2004). It has been demonstrated that the MNS is significantly activated when an individual observes or imagines a BM stimulus (Grèzes et al., 2001; Vaina et al., 2001; Servos et al., 2002; Saygin et al., 2004; Newman-Norlund et al., 2010; for reviews, see Blake and Shiffrar, 2007; Pavlova, 2012; Thompson and Parasuraman, 2012), which implies that the observers employ their own cortical motor system to simulate perceived movements and to understand the displayed stimuli (e.g., Gallese et al., 1996; Rizzolatti et al., 2001; Oberman et al., 2007). To test the MNS hypothesis, Gao et al. (2015) recruited an electroencephalograph (EEG) index of Mu-suppression, which is suggested to be closely linked to the MNS (Kuhlman, 1978; Oberman and Ramachandran, 2007; Ulloa and Pineda, 2007; Perry et al., 2010; Singh et al., 2011; Hogeveen et al., 2015). If the MNS underlies the BM maintenance, then the BM memory load should modulate the degree of Mu-suppression. Gao et al. (2015) found that Mu-suppression increased as the BM memory load increased from 2 to 4 and reached a plateau at 4 BM stimuli, which confirms the MNS prediction and is consistent with the fact that WM can store a maximum of 3–4 BM stimuli (cf. Shen et al., 2014). Moreover, the difference in Mu-suppression between memorizing 2 and 4 BM stimuli was correlated with the number of stored BM stimuli in WM; this correlation was not observed for non-BM stimuli (e.g., moving circles).

However, although Gao et al. (2015) linked the MNS to the maintenance of BM information in WM, the neural substrates supporting BM processing in WM remain unclear. Specifically, the poor spatial resolution of EEG prevents a thorough examination of the specific neural substrates for retaining BM in WM. Gao et al. (2015) also did not localize the source of Mu. Furthermore, in addition to the MNS, it has been demonstrated that the superior temporal sulcus (STS), which is a critical region in social cognition in humans (e.g., Allison et al., 2000; Keysers and Perrett, 2004; Mar, 2011; Gao et al., 2012; Lee et al., 2014), is among the core regions in charge of BM processing in perception (e.g., Saygin, 2007; Grossman et al., 2000, 2005; for reviews, see Blake and Shiffrar, 2007; Grosbras et al., 2012; Pavlova, 2012; Thompson and Parasuraman, 2012). For instance, BM stimuli induce larger STS activation compared to non-BM stimuli (e.g., scrambled or inverted BM stimuli; Grossman et al., 2000; Grèzes et al., 2001; Puce and Perrett, 2003; Saygin et al., 2004; Thompson et al., 2005; Saygin, 2007). However, Gao et al. (2015) only implied that the MNS is involved in the WM maintenance of BM. Thus, it remains unclear whether other cortical regions (particularly the STS) are also involved in BM maintenance and whether distinct brain regions are involved at different processing phases of WM, which was investigated in this study.

To this end, we adopted high spatial-resolution functional magnetic resonance imaging (fMRI) to investigate the neural substrates supporting the WM processing of BM in a change detection task, which is commonly used in WM studies (e.g., Luck and Vogel, 1997; Wheeler and Treisman, 2002; Xu, 2002; Wood, 2007, 2011; Shen et al., 2014; Gao et al., 2015, 2016). In a typical change detection task, WM processing involves at least three distinct phases: Encoding, maintenance, and retrieval (e.g., Todd and Marois, 2004; Xu and Chun, 2006, 2007). Here, we focused on the encoding and maintenance phases^1^, and used time-course analysis to differentiate distinct WM phases (e.g., Todd and Marois, 2004; Xu and Chun, 2006). We required participants to memorize BM stimuli via the PLDs technique, and examined the cortical regions that were modulated by the memory load of BM stimuli. Based on our previous EEG study of BM, which revealed that the largest load effect was observed between 2 and 4 BM stimuli (Gao et al., 2015; as is commonly used in other WM studies; e.g., Vogel and Machizawa, 2004; Gao et al., 2009; Luria and Vogel, 2011), we required participants to retain 2 or 4 BM stimuli in the task. Additionally, the BM stimuli in the memory array were selected from distinct categories with relatively long exposure times (usually at least 1 s). Under this setting, participants are likely to verbally recode the BM stimuli (e.g., labeling a BM stimulus as walking) instead of storing the motion information of the BM. To avoid this verbal strategy without impairing the storage of real BM in WM, most WM studies on BM have used a dual-task setting in which a secondary articulatory suppression task (e.g., repeating three digits throughout the course of a trial) was added to the BM memory task (e.g., Wood, 2007, 2011; Shen et al., 2014; Gao et al., 2015, 2016). It is of note that this method has also been well-accepted in WM studies (including fMRI studies) exploring other visual stimuli by assuming that the articulatory suppression task consumes resources from verbal WM and that visual stimuli require a distinct type of resource in visual WM (e.g., Vogel et al., 2001; Todd and Marois, 2004; Allen et al., 2009; see Baddeley, 2012, for a review). Our recent EEG study (Gao et al., 2015) further supports the necessity of adding a secondary verbal task to the BM memory task: Whereas significant Mu-suppression in the WM maintenance phase of BM was observed when a secondary verbal task was added, Mu-suppression vanished when the secondary task was removed. Consequently, in this study, we followed the procedures of previous WM studies of BM and required participants to covertly rehearse three numerical digits throughout the trial.

Regions of interest (ROIs) are required to conduct the time-course analysis. Because this is the first neuroimaging study to directly explore the neural substrates of BM in WM, we initially conducted a pilot study to determine possible ROIs that contribute to the WM processing of BM. We then conducted a formal experiment to measure the effects of BM load on these ROIs. Furthermore, the purpose of the current study was to reveal all potential neural substrates for holding BM in WM, including cortical regions specific to BM as well as cortical regions for processing common visual information. Therefore, we used a common fixation baseline in which participants fixated on a red cross in the center of the screen while covertly rehearsing three digits (referred to henceforth as the fixation-baseline)^2^, which has been used in previous WM studies (e.g., Todd and Marois, 2004; Xu and Chun, 2006), instead of using scrambled PLDs or inverted PLDs (for reviews see Blake and Shiffrar, 2007; Pavlova, 2012; Thompson and Parasuraman, 2012).

## Experiment for Defining ROIs

### Participants

Seventeen (11 male) neurologically normal participants (19–31 years old; 22.5 ± 3.4 years old) were paid to participate in this study. All participants were right-handed, had normal or corrected-to-normal vision, had no history of neurological or psychiatric disorders, and had not previously participated in an MRI experiment. Two male participants and one female participant were removed from further analysis because of chance level performance on the memory task. This study was approved by the Research Ethics Board of Zhejiang University and granting agency. Written informed consent was obtained from all participants in accordance with the Declaration of Helsinki.

### Stimulus and Procedure

In line with our previous BM studies of WM (Shen et al., 2014; Ding et al., 2015; Gao et al., 2015, 2016), six BM stimuli were selected from the Vanrie and Verfaillie (2004) database: Cycle, jump, paint, spade, walk, and wave. Each stimulus consisted of 30 distinct frames that were each displayed twice in succession, leading to a 1-s animation under a refresh rate of 60 Hz. Stimuli were displayed at a visual angle of approximately 1.35° × 1.35° and positioned at five potential locations that were uniformly distributed within an invisible circle (radius 4.02° of visual angle) aligned in the center of the screen.

**FIGURE 1:**
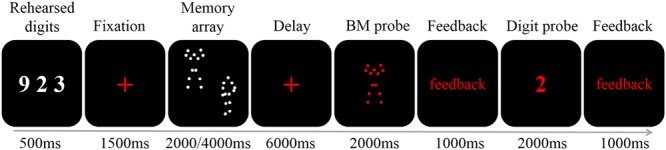
**A schematic illustration of a single trial.** Each trial began with the presentation of three digits (500 ms). After a fixation of 1500 ms two or four BM stimuli were presented for 2000 or 4000 ms, respectively. A 6000 ms fixation was followed by a 2000 ms probe. Participants judged whether the probe had appeared in the memory array, and was given feedback. Finally, the digit probe was presented. The participants responded within 2000 ms, and were given feedback.

Each trial began with three white digits presented on a black background (0, 0, 0 in RGB space) for 500 ms (**Figure 1**). Participants were instructed to rehearse these digits (e.g., by stating “nine,” “two,” and “three” instead of “nine hundred twenty-three”) covertly throughout the trial to prevent verbal coding of the stimuli (e.g., Vogel et al., 2001; Gao et al., 2015). Next, a red fixation cross (0.41° × 0.41° visual angle) was presented for 1,500 ms to prime the upcoming memory task. Then, the memory array was presented for 2,000 (load 2) or 4,000 (load 4) ms (i.e., 1 s *per* BM) to ensure participants had sufficient encoding time. Following a delay period of 6,000 ms during which a red fixation cross was presented, a red BM probe was presented in the center of the screen. Participants judged whether the BM had appeared in the memory array by pressing one of the two buttons within 2,000 ms, followed by a 1,000 ms feedback. Finally, a red digit was presented in the center of the screen for 2,000 ms, and participants had to determine whether it was among the three rehearsed digits, followed by a 1,000 ms feedback. The red colors in both the BM probe and digit probe were used to inform participants that the displayed item was the probe. Participants were instructed to respond as accurately as possible without worrying about the response time.

Additionally, after each memory-task trial there was a fixation-baseline trial, which was further followed by a 2,000 ms blank interval. For the fixation-baseline trial, three white digits were first presented for 500 ms. Participants were instructed to rehearse these digits covertly, as described above. Then, a red fixation cross was presented for 7,500 ms, during which participants continued to rehearse the digits.

The entire experiment included two runs, each with 16 pseudo-randomly presented trials (eight memory-task trials for load 2 and 4 each) and 16 fixation-baseline trials. Each run lasted 7 min and 48 s. Before the formal experiment, participants completed a practice session (16 trials) outside the scanner to become familiar with the task.

### Data Acquisition

Participants lay in a supine position inside a GE 3T scanner and viewed the display through a mirror. Foam pads were used to prevent head movement. Displays were presented via a visual and audio stimulation system for fMRI (Shenzhen Sinorad Medical Electronics Co., Ltd, Type SA-9900). Stimuli were created and presented in MATLAB (The MathWorks, Natick, MA, USA) and Psychophysics Toolbox software (Brainard, 1997). A trigger pulse from the scanner synchronized the onset of stimulus presentation at the beginning of the image acquisition.

Information about the hemodynamic response was obtained using single-shot, T2^∗^-weighted, echo-planar imaging (EPI) sequences. The acquisition parameters for the anatomical images were as follows: Repetition time (TR) = 2000 ms; echo time (TE) = 30 ms; flip angle (FA) = 90°; and field of view (FOV) = 200 mm × 200 mm. We acquired 33 axial slices (thickness = 4 mm, gap = 1 mm, in-plane resolution = 64 × 64). In addition, T1-weighted sagittal images were collected for slice localization, with acquisition parameters as follows: Short time inversion recovery (STIR) sequence; TR = 1767.42 ms; TE = 20.616 ms; FA = 90°; FOV = 200 mm × 200 mm; 33 slices, slice thickness = 4 mm, gap = 1 mm, and in-plane resolution = 512 × 512.

### fMRI Data Pre-processing

fMRI data processing and analysis were performed using Statistical Parametric Mapping (SPM) 8 (Friston et al., 1994)^3^. Functional images were corrected for slice acquisition time differences and then were corrected for head motion using a rigid body correction in SPM. None of the participants moved more than 3 mm in translational or 3° in rotational dimensions. Functional images then were co-registered with the anatomical images, which were segmented into gray matter and white matter. Anatomical images were spatially normalized to the Montreal Neurological Institute (MNI) template, and normalization parameters were applied to the functional images. Normalized images were smoothed using a Gaussian filter with a full width at half maximum of 8 mm.

### fMRI Data Analysis

Individual data were analyzed by creating a general linear model in SPM 8. For each memory load condition, three regressors were defined: Encoding [2 (for load 2) or 4 (for load 4) seconds of the memory array], maintenance (6 s of the delay after the memory array), and retrieval (2 s of the probe). Because we used two memory load conditions with different exposure times, each processing phase had two types of regressors: One for memory load 2, one for memory load 4. Additionally, there was a regressor for the fixation-baseline trial (the last 6 s of the fixation-baseline). This procedure resulted in seven regressors in total. All regressors were convolved with the canonical hemodynamic response function.

Statistical analyses were based on random-effects analysis of variance (ANOVA). To remove the effect of rehearsing digits on the blood-oxygen-level dependent (BOLD) signal of processing BM in WM, we compared the BOLD activity between the WM phases (i.e., encoding and maintenance, separately) and the fixation-baseline. Additionally, because the exposure times of the memory array were different for loads 2 and 4, we did not compare the BOLD activity between loads 2 and 4, to avoid potential confounding due to unbalanced exposure times. Instead, we conducted the comparison separately for load 2 and load 4 to obtain all possible ROIs devoted to BM processing in WM. This comparison resulted in four contrasts: Comparison between encoding for load 2 and fixation-baseline (Encoding-Two), comparison between encoding for load 4 and fixation-baseline (Encoding-Four), comparison between maintenance for load 2 and fixation-baseline (Maintenance-Two), and comparison between maintenance for load 4 and fixation-baseline (Maintenance-Four). A one-sample *t*-test was conducted for each contrast. A suprathreshold statistical map (*p*_unc_ < 0.005, uncorrected) of each condition was used to plot ROIs, which were defined by spheres with 6-mm radii centered at the maxima of each suprathreshold cluster (see **Table 1**). Each ROI contained 33 voxels.

**Table 1:** MNI coordinates of the coordinates of ROI center defined with suprathreshold cluster.

Brain region	Hemisphere	BA	Encoding two					Encoding four					Maintenance two					Maintenance four			
			*x*	*y*	*z*	*Z*-value		*x*	*y*	*z*	*Z*-value		*x*	*y*	*z*	*Z*-value		*x*	*y*	*z*	*Z*-value
**Frontal**																					
Inferior Frontal Gyrus	Right	47																36	27	-3	4.36
Middle Frontal Gyrus	Left	46																-42	27	27	5.09
	Left	9											-45	12	33	5.38		-51	24	33	4.88
	Right	9																57	24	33	4.58
	Left	6											0	12	51	5.39					
	Right	6											9	21	39	4.88					
	Right	6											3	21	48	5.32					
**Parietal**																					
Inferior Parietal Lobule	Left	40											-45	-39	48	5.80		-42	-57	57	5.13
	Left	40											-36	-51	45	5.13		-42	-42	51	4.72
Superior Parietal Lobule	Left	7	-24	-66	48	4.41		-30	-55	54	5.53										
	Right	7	33	-69	60	4.65		27	-72	42	5.88										
**Temporal**																					
Fusiform Gyrus	Left	37	-45	-60	-15	5.85		-42	-63	-18	5.58										
	Right	37	48	-54	-21	4.54															
	Right	37	45	-57	-12	4.50															
**Occipital**																					
Middle Occipital Gyrus	Left	19	-48	-72	-9	5.29															
	Right	19	48	-78	-12	4.48															

*>The *x, y, z* coordinates (in MNI space) of the maxima voxel (see *t*-value, *p*_unc_ < 0.005, uncorrected) associated with each region of the activated regions. BA, Brodmann’s.*

Accuracy on the memory task was used to examine the effect of WM load. Cowan’s formula (cf. Rouder et al., 2011) was employed to estimate the WM capacity of BM: *K* = *S* ^∗^ (*H* – *F*), where *K* is WM capacity, *S* is the number of stimuli, *H* is the hit rate, and *F* is the false alarm rate. We calculated the *K*-values for each memory load of each participant, and set the largest *K*-value (*K*_max_) as the estimated capacity of WM for each participant (e.g., Shen et al., 2014).

### Results

#### Behavioral Results

The overall accuracy of the digit task was relatively high (*M* = 0.90; *SD* = 0.12), suggesting that participants concentrated on covert digit rehearsal. For the accuracy of the BM task, a paired *t*-test revealed that the accuracy for load 2 (*M =* 0.92; *SD =* 0.10) was significantly higher than that for load 4 [*M* = 0.85; *SD* = 0.08; *t*(13) = 2.874, *p* < 0.05, Cohen’s *d* = 0.818]. By using Cowan’s formula, we found that participants could maintain 2–3 BM (*K*_max_ = 2.75) in WM.

#### fMRI Results

**FIGURE 2:**
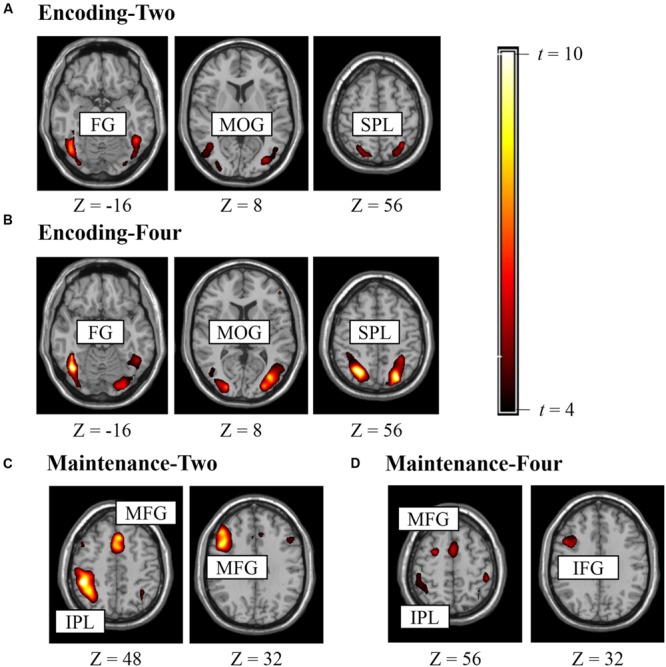
**Brain regions activated in experiment for defining ROI (*p*_unc_ < 0.005, *k* > 10). (A)** ROIs for encoding two BM stimuli, **(B)** ROIs for encoding four BM stimuli, **(C)** ROIs for retaining two BM stimuli, **(D)** ROIs for retaining four BM stimuli.

**Figure 2** shows the suprathreshold statistical map in each condition. Brain regions activated in the Encoding-Two condition included the bilateral superior parietal lobe [SPL, Brodmann Area (BA) 7], bilateral fusiform gyrus (FG, BA37), and bilateral middle occipital gyrus (MOG, BA19). Brain regions activated in the Encoding-Four condition included the bilateral SPL (BA7) and left FG (BA37). Brain regions activated in the Maintenance-Two condition included the bilateral middle frontal gyrus (MFG, BA6), left MFG (BA9), and left rIPL (BA40). Brain regions activated in the Maintenance-Four condition included the bilateral MFG (BA9), left MFG (BA46), right pIFG (BA47), and left rIPL (BA40). Please see **Table 1** for details.

Next, we combined the ROIs of the same anatomical region in each hemisphere to generate a comprehensive list of ROIs for the time-course analysis. This procedure resulted in 13 different ROIs: left MFG (BA46), right pIFG (BA47), left MFG (BA9), right MFG (BA9), left MFG (BA6), right MFG (BA6), left rIPL (BA40), left SPL (BA7), right SPL (BA7), left FG (BA37), right FG (BA37), left MOG (BA19), and right MOG (BA19). Additionally, to have a direct comparison with BM perception studies, we added two ROIs (6-mm radius) revealed by a meta-analysis as neural substrates supporting BM perception (Grosbras et al., 2012): left STS (-52, -50, 4; BA21) and left vPMC (-50, 8, 28; BA6). It should be noted that the left vPMC is embedded in the left MFG (BA6) in the current study.

## Experiment for Time-Course Analysis

### Methods

Twenty-four (12 male) neurologically normal participants (20–24 years old; 22.4 ± 2.93 years old) were paid to participate in the study. Two female participants were removed from further analysis because their level of performance on the memory task did not exceed the chance level. Two additional female participants were excluded from analysis due to a lack of time-locked fMRI signals (e.g., Todd and Marois, 2004; Xu and Chun, 2006). Therefore, the final analysis was conducted on 20 participants.

Study procedures were identical to the *experiment for defining ROIs* session, except for the aspects described below. Critically, the exposure time of the memory array was fixed at 4,000 ms for loads 2 and 4, such that the exposure time was identical during the encoding phase between the two load conditions, enabling us to directly compare BOLD activities between load 2 and 4. Additionally, the delay between the memory array and the BM probe was extended to 8,000 ms, allowing the hemodynamic response to reach its peak (e.g., Xu and Chun, 2006), and the feedback was shortened to 500 ms. The fixation-baseline was constructed by replacing the BM task in the memory-task condition (16 s in total) with a 16-s fixation while the digit rehearsal task remained (including presentation, response, and feedback for the digits). Fixation-baseline trial hence had the same duration as the memory-task trial. Following pervious fMRI studies that employed time-course analysis (e.g., Todd and Marois, 2004; Xu and Chun, 2006), we took the fixation-baseline trial as an condition, which resulted in three experimental conditions: Load 2, load 4, and fixation-baseline. There were three runs, each containing 18 pseudo-randomly presented trials (six trials each for load 2, load 4, and fixation-baseline), with a 2,000 ms blank interval between each trial. Each run lasted 6 min and 4 s. Before the formal experiment, participants completed a practice session (18 trials) outside the scanner to become familiar with the task.

For each participant, we used MarsBar ROI toolbox for SPM^4^ to extract fMRI signals from the 15 ROIs that were pre-defined in the *experiment for defining ROIs*. The signals from each ROI were segmented and averaged for each stimulus condition. These time courses then were converted to the percentage signal change for each condition by subtracting the corresponding value of the fixation-baseline and then dividing by that value (cf. Xu and Chun, 2006). Following previous ROI studies (e.g., Rypma and D’Esposito, 1999; Koshino et al., 2005; Xu and Chun, 2006), a paired *t*-test was conducted separately for the percentage signal change of each ROI with memory load as the within-subjects factor. Additionally, we also adopted Bayes factor (Jeffery–Zellner–Siow Prior Bayes factor^5^; Rouder et al., 2009) to estimate the likelihood of the null hypothesis over the alternative hypothesis.

### Results

#### Behavioral Results

The overall accuracy of the digit task was relatively high (*M* = 0.97; *SD* = 0.03), suggesting that participants concentrated on covert digit rehearsal. For the accuracy of BM memory task, a paired *t*-test revealed that the accuracy for load 2 (*M* = 0.90; *SD* = 0.08) was significantly higher than that for load 4 [*M* = 0.83; *SD* = 0.10; *t*(19) = 2.458, *p* < 0.05, Cohen’s *d* = 0.831]. By using Cowan’s formula, we found that the participants could maintain 2–3 BM (*K*_max_ = 2.71) in WM.

#### fMRI Results

**FIGURE 3:**
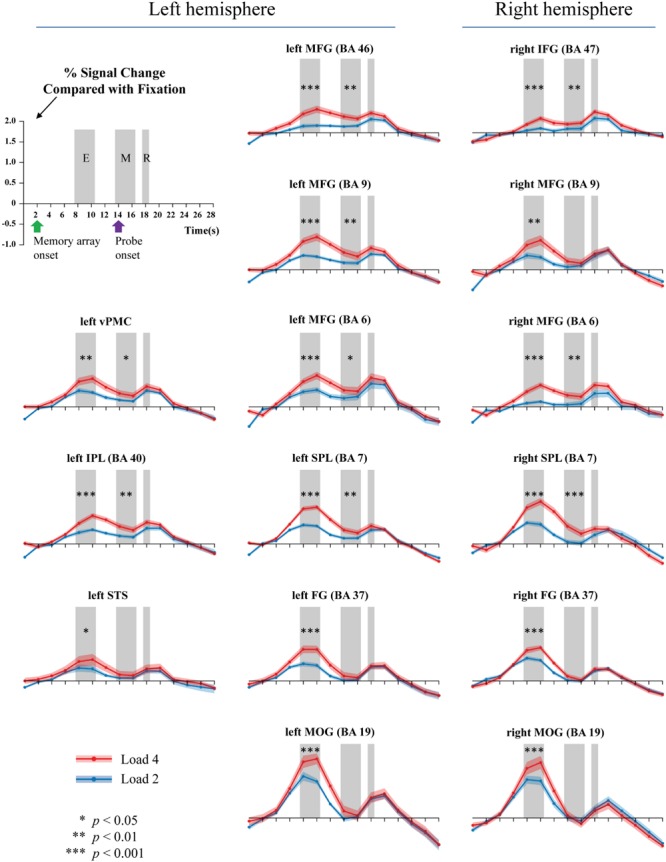
**Activations during the encoding (E), maintenance (M), and retrieval (R) phases of WM task (gray bars).** Two or four memory stimuli were presented 4000 ms. Green arrow shows the onset time of the memory array, while the purple arrow indicates the presentation of the probe.

Because the accuracy in each condition was relatively high, all trials were analyzed. **Figure 3** shows the activations extracted from the pre-defined ROIs. Two peaks were observed, corresponding to the fMRI signals for the memory onset (8–10 s) and the probe onset (18–20 s), respectively. We selected and averaged the signals between 8 and 10 s for the encoding phase, and the signals between 14 and 16 s for the maintenance phase.

**Table 2** summarizes the results. For WM encoding, all ROIs exhibited larger activation for load 4 than for load 2. For WM maintenance, only the following nine ROIs exhibited larger activation for load 4 than for load 2: Left MFG (BA6), right MFG (BA6), left MFG (BA9), left MFG (BA46), right pIFG (BA47), left SPL (BA7), right SPL (BA7), left rIPL (BA40), and left vPMC (BA6).

**Table 2:** BM load effect in WM encoding and maintenance.

Region	Hemisphere	BA	Load effect for encoding		Load effect for maintenance	
			*t*	Bayes factor	*t*	Bayes factor
**Frontal**						
Inferior Frontal Gyrus	Right	47	5.118^∗∗∗^	0.002	3.146^∗∗^	0.115
Middle Frontal Gyrus	Left	46	5.595^∗∗∗^	0.001	3.594^∗∗^	0.048
	Left	9	5.564^∗∗∗^	0.001	3.084^∗∗^	0.129
	Right	9	3.413^∗∗^	0.069	1.215	2.260
	Left	6	6.471^∗∗∗^	1.683 × 10^-4^	2.831^∗^	0.206
	Right	6	7.687^∗∗∗^	1.859 × 10^-5^	3.794^∗∗^	0.033
Ventral Premotor Cortex	Left	6	3.768^∗∗^	0.034	2.236^∗^	0.574
**Parietal**						
Inferior Parietal Lobule	Left	40	5.369^∗∗∗^	0.001	3.431^∗∗^	0.067
Superior Parietal Lobule	Left	7	10.021^∗∗∗^	4.260 × 10^-7^	3.638^∗∗^	0.044
	Right	7	8.180^∗∗∗^	7.970 × 10^-6^	4.786^∗∗∗^	0.004
**Temporal**						
Fusiform Gyrus	Left	37	4.691^∗∗∗^	0.005	0.988	2.796
	Right	37	6.339^∗∗∗^	2.158 × 10^-4^	1.388	1.874
Superior Temporal Sulcus	Left	21	2.269^∗^	0.544	0.899	3.007
**Occipital**						
Middle Occipital Gyrus	Left	19	4.088^∗∗^	0.018	0.884	3.042
	Right	19	4.842^∗∗∗^	0.004	0.107	4.281

*BA, Brodmann’s area. ^∗^*p* < 0.05; ^∗∗^*p* < 0.01; ^∗∗∗^*p* < 0.001. Here the Bayes factor measures the how much more likely is the null hypothesis compared to the alternative hypothesis.*

## Discussion

To the best of our knowledge, this is the first study to directly investigate the neural substrates underlying BM processing in WM by focusing on the WM encoding and maintenance phases. To fully understand the neural substrates underlying BM storage in WM, the current study followed the procedures outlined in Xu and Chun (2006) by comparing activation during the encoding and maintenance phases to a fixation-baseline instead of a scrambled PLD. To directly compare to previous BM perception studies, we also included two other ROIs (STS and vPMC), which are suggested as the neural substrates underlying BM perception (Grosbras et al., 2012). We found that all activations in the pre-defined 15 ROIs were positively modulated by the memory load of BM in the WM encoding phase, suggesting that these areas were involved in the encoding of visual information conveyed by the BM. Moreover, most of these pre-defined ROIs were also contributed to the maintenance of BM information in WM, including the left MFG (BA6), right MFG (BA6), left MFG (BA9), left MFG (BA46), right pIFG (BA47), left SPL (BA7), right SPL (BA7), left rIPL (BA40), and left vPMC (BA6).

We found that the classic constituent regions of MNS, which have been revealed in previous BM perception studies, were involved in both WM encoding and WM maintenance of BM. These results are consistent with previous studies showing that the MNS is dedicated to BM processing (e.g., Rizzolatti and Craighero, 2004; Saygin et al., 2004; Grosbras et al., 2012; Pavlova, 2012; Thompson and Parasuraman, 2012), and also extend the function of the MNS from BM perception to WM. The results of the current study are also congruent with the EEG findings in Gao et al. (2015), as the current study provides direct fMRI evidence that the MNS is indeed devoted to the rehearsal of BM information in WM. Therefore, both EEG and fMRI evidence suggest that we retain BM stimuli by simulating BM via our cortical motor system (Cook et al., 2014). The current study also offers the first fMRI evidence in support of the “theory of event coding,” (Hommel et al., 2001; see Prinz, 1997 for a similar claim) which predicts that the same mental representations are employed when memorizing an action as when executing that same action. Additionally, the current finding provides indirect evidence that the EEG Mu-suppression signal has a close relationship with the MNS (Kuhlman, 1978; Oberman and Ramachandran, 2007; Ulloa and Pineda, 2007; Perry et al., 2010; Singh et al., 2011; Hogeveen et al., 2015). Moreover, it is worthwhile to note that, except for the vPMC (BA6), other regions of the MFG were also activated. These extra regions of the MFG may be related to general visual processing involved in BM (see discussion below).

The STS is a key region in BM perception (Pavlova, 2012). We found that the memory load is significantly modulated by STS activation in WM encoding but not in WM maintenance. Previous neuroimaging studies have suggested that the STS integrates information derived from both the ventral ‘what’ and the dorsal ‘where’ visual pathways to construct a high-level representation of actions (for review, see Blake and Shiffrar, 2007), which provide the motoric aspects of the action to be imitated by the MNS (for review, see Lacoboni and Dapretto, 2006). In the current WM encoding phase, BM perception must occur in order to construct integrated BM percepts; therefore, it is reasonable to find that STS activation was modulated by the memory load. Based on these findings, however, it remains unclear why STS activation was not observed during the WM maintenance phase. There are at least two explanations for this finding. First, it is possible that the role of STS in BM integration during the encoding phase negates the need for STS activation during the maintenance phase. Given that the MNS is employed to rehearse the integrated BM (cf. Gao et al., 2015; and the aforementioned MNS results), the STS may not be needed for reconstruction of the BM. This possibility is somewhat supported by our findings. In particular, we found that the FG and MOG exhibited a similar pattern to the STS such that there was a load effect on these regions during the encoding phase but not during the maintenance phase. Both the FG and MOG have been implicated in the processing of lower-level information prior to STS during BM perception (Grosbras et al., 2012; for review, see Blake and Shiffrar, 2007; Thompson and Parasuraman, 2012). Second, it is possible that our design/analysis was not sensitive enough to reveal the involvement of the STS during the WM maintenance phase. Most previous neuroimaging studies investigating BM used scrambled or inverted PLDs as a baseline (for reviews, see Blake and Shiffrar, 2007; Pavlova, 2012; Thompson and Parasuraman, 2012; Troje, 2013), which are comprised of individual dot trajectories identical to those of intact PLDs but with global, spatiotemporal coherence to depict human action. This method is appropriate for locating specific brain regions specialized for processing the kinematics that define BM. In contrast, the current study used a fixation-baseline in order to observe all possible neural substrates underlying BM processing in WM. Although our method inevitably led to the identification of more activated regions, this does not necessarily mean that this method is more sensitive in revealing STS activation. Further studies are needed to elucidate this issue. If the STS indeed does not play a role in WM maintenance, it would shed an important light on understanding the function of STS and the rehearsal mechanisms of BM. Additionally, it should be noted that we did not find significant STS activations during the encoding phase in *Experiment for defining ROIs*. One possible reason is that the imperfect parameters adopted in that experiment (e.g., the unbalanced exposure time of the memory array between loads 2 and 4, and low power from the relatively few trials per subject) may have reduced the power to reveal STS activation. We found significant STS activation after controlling for these factors in our time-course analysis experiment, which is consistent with this possibility.

Additionally, due to the specific baseline we adopted, we found that the bilateral SPL and bilateral prefrontal cortex (i.e., BA9 and BA46) were significantly modulated by the memory load of BM in both the encoding and maintenance phase. These regions are routinely found to be involved in WM tasks. As to the SPL, previous WM studies using common visual stimuli found a strong load effect in this region, suggesting that the SPL plays a pivotal role in retaining visual information in WM (e.g., Todd and Marois, 2004; Xu and Chun, 2006; Gao et al., 2011). As to the prefrontal cortex, it plays a pivotal role in WM processing, including attention control and information manipulation (e.g., D’Esposito et al., 1995; Curtis and D’Esposito, 2003; Barbey et al., 2013). Therefore, we consider activation of the SPL and prefrontal cortex to reflect general visual processing of BM stimuli. From this perspective, this finding implies that perhaps there are two distinct rehearsal mechanisms for BM: One that is specific to BM information and employs the MNS system and another that employs common visual regions such as the SPL to retain general visual information. It will be interesting to verify this possibility and explore how these two distinct mechanisms interact in future studies.

Finally, the largely overlapping regions for WM encoding and maintenance in our current study adds to evidence that WM and perception recruit similar neural mechanisms. However, distinct from previous studies using static simple stimuli (e.g., Gabor patches; Ester et al., 2009; Harrison and Tong, 2009), the PLDs used are far more complex and convey rich social information. Therefore, it seems that the shared neural mechanism between perception and WM is a general mechanism in our brain. However, it is important to note that the current study also identified at least three regions dedicated to BM encoding, implying that there may be certain processing differences between the perception and WM (Offen et al., 2009).

## Author Contributions

ZG, XL, JH, and MS initiated the conception and designed the study; XL, JH, YY, XW, and ZG, performed data acquisition, analysis, and interpretation; XL, JH, YY, MS, and ZG drafted the manuscript, XL, JH, YY, MS, WX, and ZG revised the manuscript together.

## Conflict of Interest Statement

The authors declare that the research was conducted in the absence of any commercial or financial relationships that could be construed as a potential conflict of interest.

## Footnotes

1 The retrieval phase was not analyzed in this study because activation during this phase are masked by the responses.

2 Digit rehearsal inevitably evoked certain brain activations that may confound the activations of BM. However, we required participants to rehearse the digits during the fixation-baseline in an attempt to minimize these brain activations when comparing to the results of the WM task.

3 www.fil.ion.ucl.ac.uk/spm/

4 http://marsbar.sourceforge.net/

5 http://pcl.missouri.edu/bayesfactor
